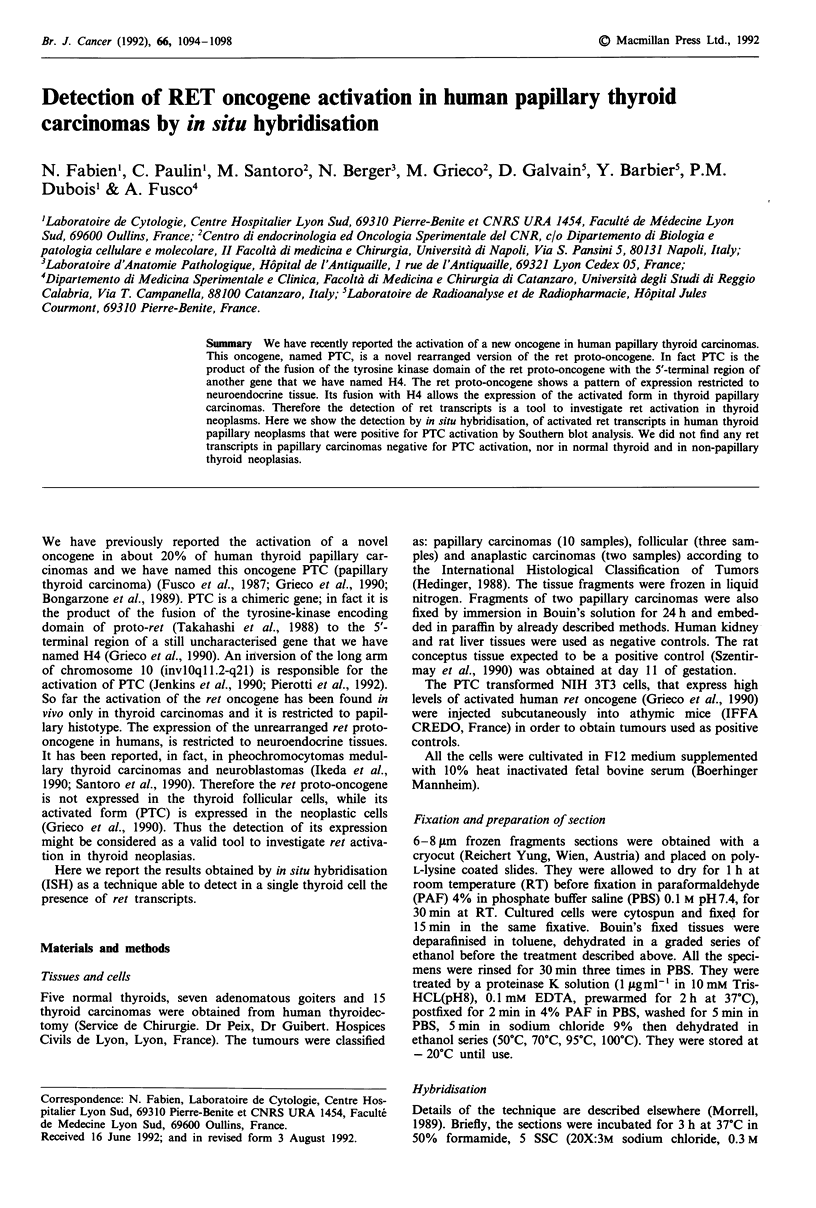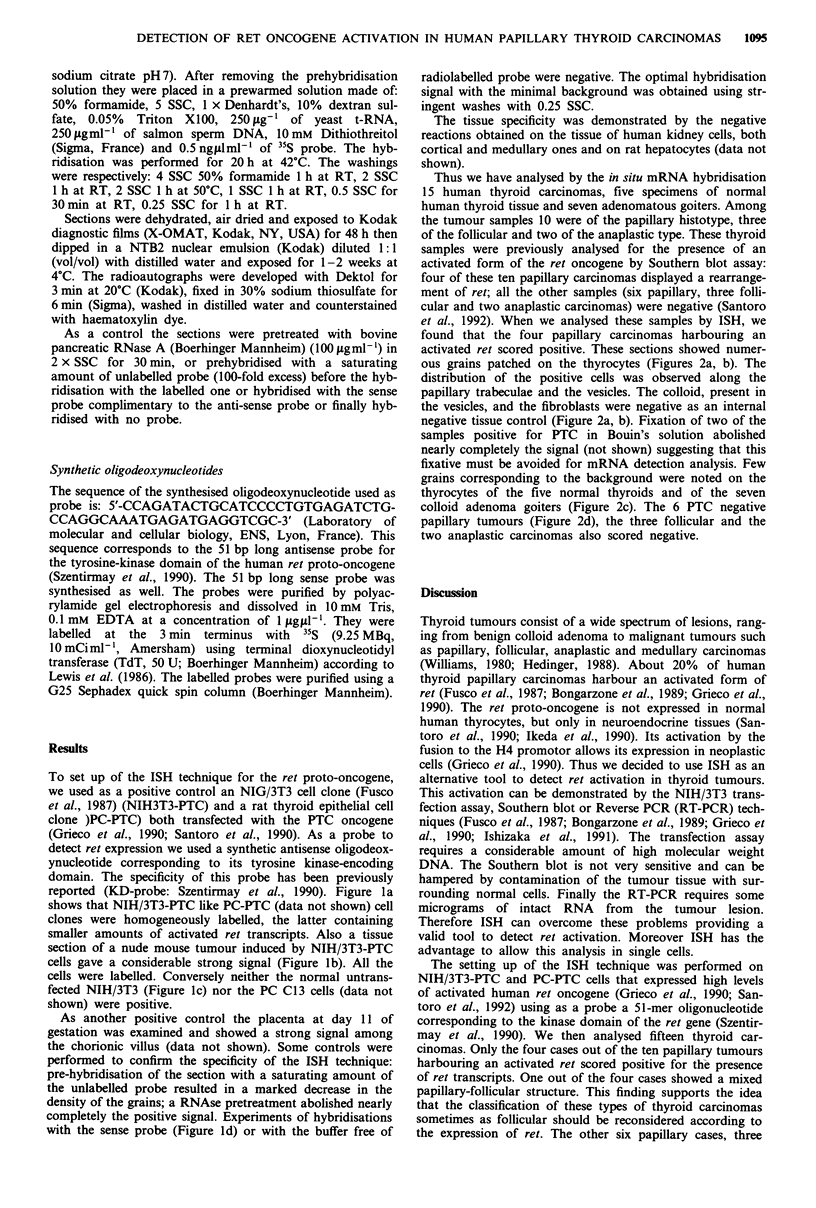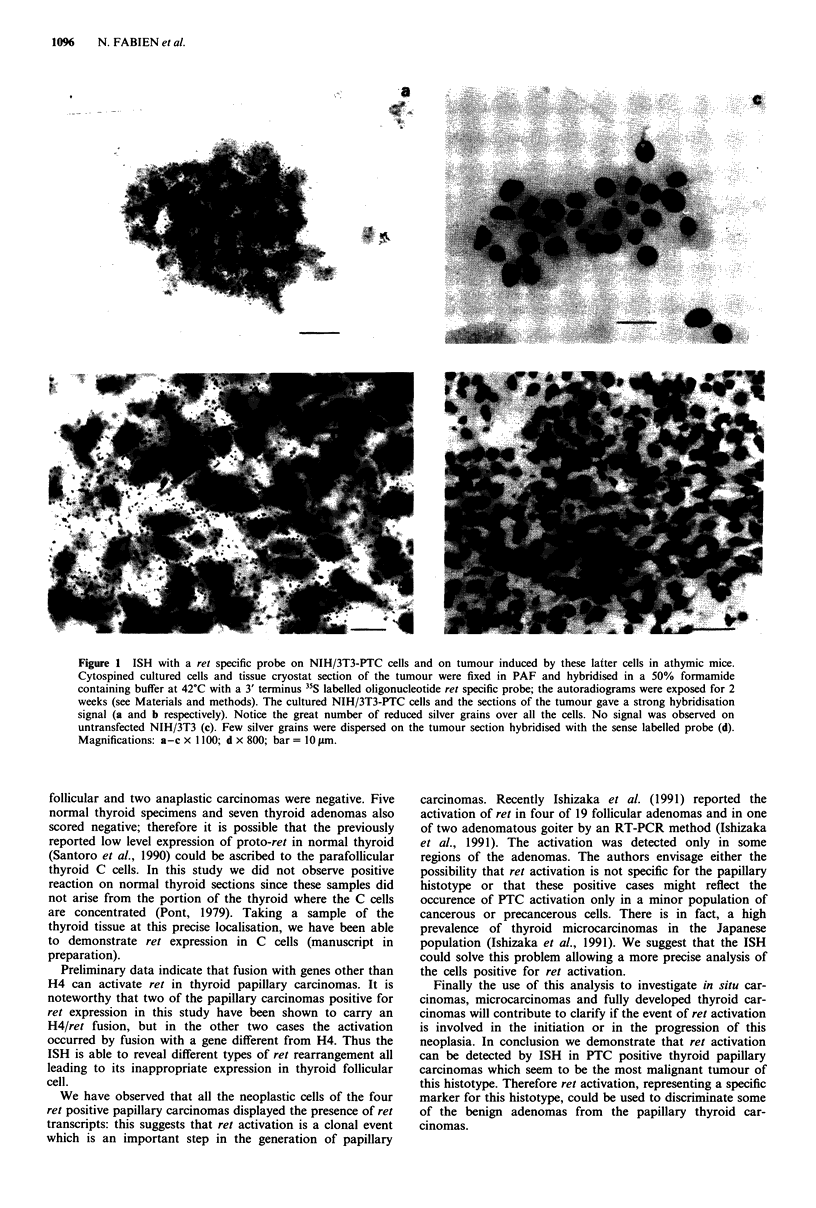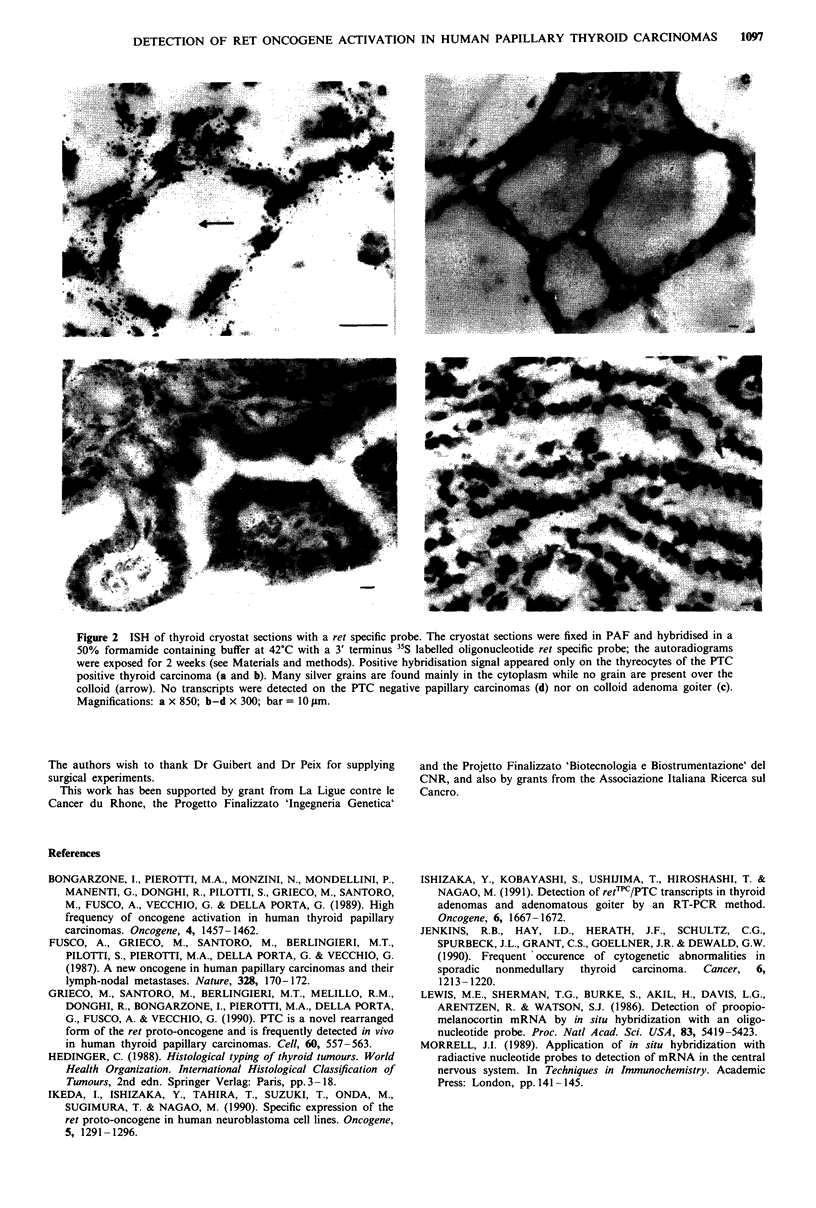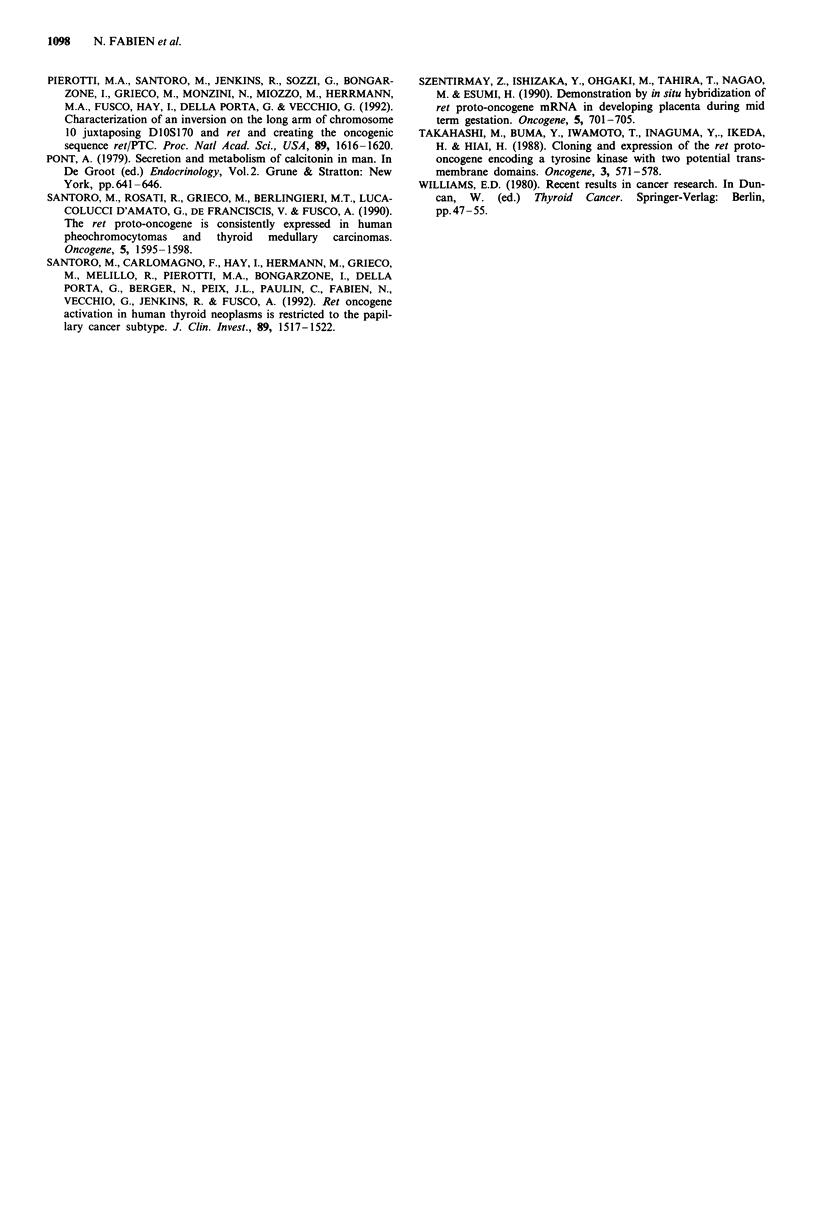# Detection of RET oncogene activation in human papillary thyroid carcinomas by in situ hybridisation.

**DOI:** 10.1038/bjc.1992.416

**Published:** 1992-12

**Authors:** N. Fabien, C. Paulin, M. Santoro, N. Berger, M. Grieco, D. Galvain, Y. Barbier, P. M. Dubois, A. Fusco

**Affiliations:** Laboratoire de Cytologie, Centre Hospitalier Lyon Sud, Oullins, France.

## Abstract

**Images:**


Br. J. Cancer (1992), 66, 1094–1098 © Macmillan Press Ltd., 1992

### Detection of RET oncogene activation in human papillary thyroid carcinomas by *in situ* hybridisation

N. Fabien[1], C. Paulin[1], M. Santoro[2], N. Berger[3], M. Grieco[2], D. Galvain[5], Y. Barbier[5], P.M. Dubois[1] & A. Fusco[4]

[1]*Laboratoire de Cytologie, Centre Hospitalier Lyon Sud, 69310 Pierre-Benite et CNRS URA 1454, Faculté de Médecine Lyon Sud, 69600 Oullins, France;* [2]*Centro di endocrinologia ed Oncologia Sperimentale del CNR, c/o Dipartemento di Biologia e patologia cellulare e molecolare, II Facoltà di medicina e Chirurgia, Università di Napoli, Via S. Pansini 5, 80131 Napoli, Italy;* [3]*Laboratoire d'Anatomie Pathologique, Hôpital de l'Antiquaille, 1 rue de l'Antiquaille, 69321 Lyon Cedex 05, France;* [4]*Dipartemento di Medicina Sperimentale e Clinica, Facoltà di Medicina e Chirurgia di Catanzaro, Università degli Studi di Reggio Calabria, Via T. Campanella, 88100 Catanzaro, Italy;* [5]*Laboratoire de Radioanalyse et de Radiopharmacie, Hôpital Jules Courmont, 69310 Pierre-Benite, France.*

**Summary** We have recently reported the activation of a new oncogene in human papillary thyroid carcinomas. This oncogene, named PTC, is a novel rearranged version of the ret proto-oncogene. In fact PTC is the product of the fusion of the tyrosine kinase domain of the ret proto-oncogene with the 5′-terminal region of another gene that we have named H4. The ret proto-oncogene shows a pattern of expression restricted to neuroendocrine tissue. Its fusion with H4 allows the expression of the activated form in thyroid papillary carcinomas. Therefore the detection of ret transcripts is a tool to investigate ret activation in thyroid neoplasms. Here we show the detection by *in situ* hybridisation, of activated ret transcripts in human thyroid papillary neoplasms that were positive for PTC activation by Southern blot analysis. We did not find any ret transcripts in papillary carcinomas negative for PTC activation, nor in normal thyroid and in non-papillary thyroid neoplasias.

We have previously reported the activation of a novel oncogene in about 20% of human thyroid papillary carcinomas and we have named this oncogene PTC (papillary thyroid carcinoma) (Fusco *et al.*, 1987; Grieco *et al.*, 1990; Bongarzone *et al.*, 1989). PTC is a chimeric gene; in fact it is the product of the fusion of the tyrosine-kinase encoding domain of proto-*ret* (Takahashi *et al.*, 1988) to the 5′-terminal region of a still uncharacterised gene that we have named H4 (Grieco *et al.*, 1990). An inversion of the long arm of chromosome 10 (inv10q11.2-q21) is responsible for the activation of PTC (Jenkins *et al.*, 1990; Pierotti *et al.*, 1992). So far the activation of the *ret* oncogene has been found *in vivo* only in thyroid carcinomas and it is restricted to papillary histotype. The expression of the unrearranged *ret* proto-oncogene in humans, is restricted to neuroendocrine tissues. It has been reported, in fact, in pheochromocytomas medullary thyroid carcinomas and neuroblastomas (Ikeda *et al.*, 1990; Santoro *et al.*, 1990). Therefore the *ret* proto-oncogene is not expressed in the thyroid follicular cells, while its activated form (PTC) is expressed in the neoplastic cells (Grieco *et al.*, 1990). Thus the detection of its expression might be considered as a valid tool to investigate *ret* activation in thyroid neoplasias.

Here we report the results obtained by *in situ* hybridisation (ISH) as a technique able to detect in a single thyroid cell the presence of *ret* transcripts.

### Materials and methods

### *Tissues and cells*

Five normal thyroids, seven adenomatous goiters and 15 thyroid carcinomas were obtained from human thyroidectomy (Service de Chirurgie. Dr Peix, Dr Guibert. Hospices Civils de Lyon, Lyon, France). The tumours were classified as: papillary carcinomas (10 samples), follicular (three samples) and anaplastic carcinomas (two samples) according to the International Histological Classification of Tumors (Hedinger, 1988). The tissue fragments were frozen in liquid nitrogen. Fragments of two papillary carcinomas were also fixed by immersion in Bouin's solution for 24 h and embedded in paraffin by already described methods. Human kidney and rat liver tissues were used as negative controls. The rat conceptus tissue expected to be a positive control (Szentirmay *et al.*, 1990) was obtained at day 11 of gestation.

The PTC transformed NIH 3T3 cells, that express high levels of activated human *ret* oncogene (Grieco *et al.*, 1990) were injected subcutaneously into athymic mice (IFFA CREDO, France) in order to obtain tumours used as positive controls.

All the cells were cultivated in F12 medium supplemented with 10% heat inactivated fetal bovine serum (Boerhinger Mannheim).

### *Fixation and preparation of section*

6–8 μm frozen fragments sections were obtained with a cryocut (Reichert Yung, Wien, Austria) and placed on poly-L-lysine coated slides. They were allowed to dry for 1 h at room temperature (RT) before fixation in paraformaldehyde (PAF) 4% in phosphate buffer saline (PBS) 0.1 M pH 7.4, for 30 min at RT. Cultured cells were cytospun and fixed for 15 min in the same fixative. Bouin's fixed tissues were deparafinised in toluene, dehydrated in a graded series of ethanol before the treatment described above. All the specimens were rinsed for 30 min three times in PBS. They were treated by a proteinase K solution (1 $\mu g ml^{-1}$ in 10 mM Tris-HCL(pH8), 0.1 mM EDTA, prewarmed for 2 h at 37°C), postfixed for 2 min in 4% PAF in PBS, washed for 5 min in PBS, 5 min in sodium chloride 9% then dehydrated in ethanol series (50°C, 70°C, 95°C, 100°C). They were stored at − 20°C until use.

### *Hybridisation*

Details of the technique are described elsewhere (Morrell, 1989). Briefly, the sections were incubated for 3 h at 37°C in 50% formamide, 5 SSC (20X:3M sodium chloride, 0.3 M

Correspondence: N. Fabien, Laboratoire de Cytologie, Centre Hospitalier Lyon Sud, 69310 Pierre-Benite et CNRS URA 1454, Faculté de Medecine Lyon Sud, 69600 Oullins, France.
Received 16 June 1992; and in revised form 3 August 1992.

sodium citrate pH7). After removing the prehybridisation solution they were placed in a prewarmed solution made of: 50% formamide, 5 SSC, 1 × Denhardt's, 10% dextran sulfate, 0.05% Triton X100, 250 $\mu g^{-1}$ of yeast t-RNA, 250 $\mu g ml^{-1}$ of salmon sperm DNA, 10 mM Dithiothreitol (Sigma, France) and 0.5 ng$\mu$l ml$^{-1}$ of $^{35}S$ probe. The hybridisation was performed for 20 h at 42°C. The washings were respectively: 4 SSC 50% formamide 1 h at RT, 2 SSC 1 h at RT, 2 SSC 1 h at 50°C, 1 SSC 1 h at RT, 0.5 SSC for 30 min at RT, 0.25 SSC for 1 h at RT.

Sections were dehydrated, air dried and exposed to Kodak diagnostic films (X-OMAT, Kodak, NY, USA) for 48 h then dipped in a NTB2 nuclear emulsion (Kodak) diluted 1:1 (vol/vol) with distilled water and exposed for 1–2 weeks at 4°C. The radioautographs were developed with Dektol for 3 min at 20°C (Kodak), fixed in 30% sodium thiosulfate for 6 min (Sigma), washed in distilled water and counterstained with haematoxylin dye.

As a control the sections were pretreated with bovine pancreatic RNase A (Boerhinger Mannheim) (100 $\mu g ml^{-1}$) in 2 × SSC for 30 min, or prehybridised with a saturating amount of unlabelled probe (100-fold excess) before the hybridisation with the labelled one or hybridised with the sense probe complimentary to the anti-sense probe or finally hybridised with no probe.

### Synthetic oligodeoxynucleotides

The sequence of the synthesised oligodeoxynucleotide used as probe is: 5'-CCAGATACTGCATCCCCTGTGAGATCTG-CCAGGCAAATGAGATGAGGTCGC-3' (Laboratory of molecular and cellular biology, ENS, Lyon, France). This sequence corresponds to the 51 bp long antisense probe for the tyrosine-kinase domain of the human *ret* proto-oncogene (Szentirmay *et al.*, 1990). The 51 bp long sense probe was synthesised as well. The probes were purified by polyacrylamide gel electrophoresis and dissolved in 10 mM Tris, 0.1 mM EDTA at a concentration of 1 $\mu g \mu l^{-1}$. They were labelled at the 3 min terminus with $^{35}S$ (9.25 MBq, 10 mCi ml$^{-1}$, Amersham) using terminal dioxynucleotidyl transferase (TdT, 50 U; Boerhinger Mannheim) according to Lewis *et al.* (1986). The labelled probes were purified using a G25 Sephadex quick spin column (Boerhinger Mannheim).

### Results

To set up of the ISH technique for the *ret* proto-oncogene, we used as a positive control an NIG/3T3 cell clone (Fusco *et al.*, 1987) (NIH3T3-PTC) and a rat thyroid epithelial cell clone )PC-PTC) both transfected with the PTC oncogene (Grieco *et al.*, 1990; Santoro *et al.*, 1990). As a probe to detect *ret* expression we used a synthetic antisense oligodeoxynucleotide corresponding to its tyrosine kinase-encoding domain. The specificity of this probe has been previously reported (KD-probe: Szentirmay *et al.*, 1990). Figure 1a shows that NIH/3T3-PTC like PC-PTC (data not shown) cell clones were homogeneously labelled, the latter containing smaller amounts of activated *ret* transcripts. Also a tissue section of a nude mouse tumour induced by NIH/3T3-PTC cells gave a considerable strong signal (Figure 1b). All the cells were labelled. Conversely neither the normal untransfected NIH/3T3 (Figure 1c) nor the PC C13 cells (data not shown) were positive.

As another positive control the placenta at day 11 of gestation was examined and showed a strong signal among the chorionic villus (data not shown). Some controls were performed to confirm the specificity of the ISH technique: pre-hybridisation of the section with a saturating amount of the unlabelled probe resulted in a marked decrease in the density of the grains; a RNAse pretreatment abolished nearly completely the positive signal. Experiments of hybridisations with the sense probe (Figure 1d) or with the buffer free of radiolabelled probe were negative. The optimal hybridisation signal with the minimal background was obtained using stringent washes with 0.25 SSC.

The tissue specificity was demonstrated by the negative reactions obtained on the tissue of human kidney cells, both cortical and medullary ones and on rat hepatocytes (data not shown).

Thus we have analysed by the *in situ* mRNA hybridisation 15 human thyroid carcinomas, five specimens of normal human thyroid tissue and seven adenomatous goiters. Among the tumour samples 10 were of the papillary histotype, three of the follicular and two of the anaplastic type. These thyroid samples were previously analysed for the presence of an activated form of the *ret* oncogene by Southern blot assay: four of these ten papillary carcinomas displayed a rearrangement of *ret*; all the other samples (six papillary, three follicular and two anaplastic carcinomas) were negative (Santoro *et al.*, 1992). When we analysed these samples by ISH, we found that the four papillary carcinomas harbouring an activated *ret* scored positive. These sections showed numerous grains patched on the thyrocytes (Figures 2a, b). The distribution of the positive cells was observed along the papillary trabeculae and the vesicles. The colloid, present in the vesicles, and the fibroblasts were negative as an internal negative tissue control (Figure 2a, b). Fixation of two of the samples positive for PTC in Bouin's solution abolished nearly completely the signal (not shown) suggesting that this fixative must be avoided for mRNA detection analysis. Few grains corresponding to the background were noted on the thyrocytes of the five normal thyroids and of the seven colloid adenoma goiters (Figure 2c). The 6 PTC negative papillary tumours (Figure 2d), the three follicular and the two anaplastic carcinomas also scored negative.

### Discussion

Thyroid tumours consist of a wide spectrum of lesions, ranging from benign colloid adenoma to malignant tumours such as papillary, follicular, anaplastic and medullary carcinomas (Williams, 1980; Hedinger, 1988). About 20% of human thyroid papillary carcinomas harbour an activated form of *ret* (Fusco *et al.*, 1987; Bongarzone *et al.*, 1989; Grieco *et al.*, 1990). The *ret* proto-oncogene is not expressed in normal human thyrocytes, but only in neuroendocrine tissues (Santoro *et al.*, 1990; Ikeda *et al.*, 1990). Its activation by the fusion to the H4 promotor allows its expression in neoplastic cells (Grieco *et al.*, 1990). Thus we decided to use ISH as an alternative tool to detect *ret* activation in thyroid tumours. This activation can be demonstrated by the NIH/3T3 transfection assay, Southern blot or Reverse PCR (RT-PCR) techniques (Fusco *et al.*, 1987; Bongarzone *et al.*, 1989; Grieco *et al.*, 1990; Ishizaka *et al.*, 1991). The transfection assay requires a considerable amount of high molecular weight DNA. The Southern blot is not very sensitive and can be hampered by contamination of the tumour tissue with surrounding normal cells. Finally the RT-PCR requires some micrograms of intact RNA from the tumour lesion. Therefore ISH can overcome these problems providing a valid tool to detect *ret* activation. Moreover ISH has the advantage to allow this analysis in single cells.

The setting up of the ISH technique was performed on NIH/3T3-PTC and PC-PTC cells that expressed high levels of activated human *ret* oncogene (Grieco *et al.*, 1990; Santoro *et al.*, 1992) using as a probe a 51-mer oligonucleotide corresponding to the kinase domain of the *ret* gene (Szentirmay *et al.*, 1990). We then analysed fifteen thyroid carcinomas. Only the four cases out of the ten papillary tumours harbouring an activated *ret* scored positive for the presence of *ret* transcripts. One out of the four cases showed a mixed papillary-follicular structure. This finding supports the idea that the classification of these types of thyroid carcinomas sometimes as follicular should be reconsidered according to the expression of *ret*. The other six papillary cases, three

**Figure 1** ISH with a *ret* specific probe on NIH/3T3-PTC cells and on tumour induced by these latter cells in athymic mice. Cytospined cultured cells and tissue cryostat section of the tumour were fixed in PAF and hybridised in a 50% formamide containing buffer at 42°C with a 3′ terminus $^{35}S$ labelled oligonucleotide *ret* specific probe; the autoradiograms were exposed for 2 weeks (see Materials and methods). The cultured NIH/3T3-PTC cells and the sections of the tumour gave a strong hybridisation signal (**a** and **b** respectively). Notice the great number of reduced silver grains over all the cells. No signal was observed on untransfected NIH/3T3 (**c**). Few silver grains were dispersed on the tumour section hybridised with the sense labelled probe (**d**). Magnifications: **a**–**c** × 1100; **d** × 800; bar = 10 µm.

follicular and two anaplastic carcinomas were negative. Five normal thyroid specimens and seven thyroid adenomas also scored negative; therefore it is possible that the previously reported low level expression of proto-*ret* in normal thyroid (Santoro *et al.*, 1990) could be ascribed to the parafollicular thyroid C cells. In this study we did not observe positive reaction on normal thyroid sections since these samples did not arise from the portion of the thyroid where the C cells are concentrated (Pont, 1979). Taking a sample of the thyroid tissue at this precise localisation, we have been able to demonstrate *ret* expression in C cells (manuscript in preparation).

Preliminary data indicate that fusion with genes other than H4 can activate *ret* in thyroid papillary carcinomas. It is noteworthy that two of the papillary carcinomas positive for *ret* expression in this study have been shown to carry an H4/*ret* fusion, but in the other two cases the activation occurred by fusion with a gene different from H4. Thus the ISH is able to reveal different types of *ret* rearrangement all leading to its inappropriate expression in thyroid follicular cell.

We have observed that all the neoplastic cells of the four *ret* positive papillary carcinomas displayed the presence of *ret* transcripts: this suggests that *ret* activation is a clonal event which is an important step in the generation of papillary carcinomas. Recently Ishizaka *et al.* (1991) reported the activation of *ret* in four of 19 follicular adenomas and in one of two adenomatous goiter by an RT-PCR method (Ishizaka *et al.*, 1991). The activation was detected only in some regions of the adenomas. The authors envisage either the possibility that *ret* activation is not specific for the papillary histotype or that these positive cases might reflect the occurence of PTC activation only in a minor population of cancerous or precancerous cells. There is in fact, a high prevalence of thyroid microcarcinomas in the Japanese population (Ishizaka *et al.*, 1991). We suggest that the ISH could solve this problem allowing a more precise analysis of the cells positive for *ret* activation.

Finally the use of this analysis to investigate *in situ* carcinomas, microcarcinomas and fully developed thyroid carcinomas will contribute to clarify if the event of *ret* activation is involved in the initiation or in the progression of this neoplasia. In conclusion we demonstrate that *ret* activation can be detected by ISH in PTC positive thyroid papillary carcinomas which seem to be the most malignant tumour of this histotype. Therefore *ret* activation, representing a specific marker for this histotype, could be used to discriminate some of the benign adenomas from the papillary thyroid carcinomas.

**Figure 2** ISH of thyroid cryostat sections with a *ret* specific probe. The cryostat sections were fixed in PAF and hybridised in a 50% formamide containing buffer at 42°C with a 3′ terminus $^{35}S$ labelled oligonucleotide *ret* specific probe; the autoradiograms were exposed for 2 weeks (see Materials and methods). Positive hybridisation signal appeared only on the thyreocytes of the PTC positive thyroid carcinoma (**a** and **b**). Many silver grains are found mainly in the cytoplasm while no grain are present over the colloid (arrow). No transcripts were detected on the PTC negative papillary carcinomas (**d**) nor on colloid adenoma goiter (**c**). Magnifications: **a** × 850; **b**–**d** × 300; bar = 10 µm.

The authors wish to thank Dr Guibert and Dr Peix for supplying surgical experiments.

This work has been supported by grant from La Ligue contre le Cancer du Rhone, the Progetto Finalizzato 'Ingegneria Genetica' and the Projetto Finalizzato 'Biotecnologia e Biostrumentazione' del CNR, and also by grants from the Associazione Italiana Ricerca sul Cancro.

### References

BONGARZONE, I., PIEROTTI, M.A., MONZINI, N., MONDELLINI, P., MANENTI, G., DONGHI, R., PILOTTI, S., GRIECO, M., SANTORO, M., FUSCO, A., VECCHIO, G. & DELLA PORTA, G. (1989). High frequency of oncogene activation in human thyroid papillary carcinomas. *Oncogene*, **4**, 1457–1462.

FUSCO, A., GRIECO, M., SANTORO, M., BERLINGIERI, M.T., PILOTTI, S., PIEROTTI, M.A., DELLA PORTA, G. & VECCHIO, G. (1987). A new oncogene in human papillary carcinomas and their lymph-nodal metastases. *Nature*, **328**, 170–172.

GRIECO, M., SANTORO, M., BERLINGIERI, M.T., MELILLO, R.M., DONGHI, R., BONGARZONE, I., PIEROTTI, M.A., DELLA PORTA, G., FUSCO, A. & VECCHIO, G. (1990). PTC is a novel rearranged form of the *ret* proto-oncogene and is frequently detected *in vivo* in human thyroid papillary carcinomas. *Cell*, **60**, 557–563.

HEDINGER, C. (1988). *Histological typing of thyroid tumours. World Health Organization. International Histological Classification of Tumours*, 2nd edn. Springer Verlag: Paris, pp. 3–18.

IKEDA, I., ISHIZAKA, Y., TAHIRA, T., SUZUKI, T., ONDA, M., SUGIMURA, T. & NAGAO, M. (1990). Specific expression of the *ret* proto-oncogene in human neuroblastoma cell lines. *Oncogene*, **5**, 1291–1296.

ISHIZAKA, Y., KOBAYASHI, S., USHIJIMA, T., HIROSHASHI, T. & NAGAO, M. (1991). Detection of $ret^{TPC}$/PTC transcripts in thyroid adenomas and adenomatous goiter by an RT-PCR method. *Oncogene*, **6**, 1667–1672.

JENKINS, R.B., HAY, I.D., HERATH, J.F., SCHULTZ, C.G., SPURBECK, J.L., GRANT, C.S., GOELLNER, J.R. & DEWALD, G.W. (1990). Frequent occurence of cytogenetic abnormalities in sporadic nonmedullary thyroid carcinoma. *Cancer*, **6**, 1213–1220.

LEWIS, M.E., SHERMAN, T.G., BURKE, S., AKIL, H., DAVIS, L.G., ARENTZEN, R. & WATSON, S.J. (1986). Detection of proopiomelanocortin mRNA by *in situ* hybridization with an oligonucleotide probe. *Proc. Natl Acad. Sci. USA*, **83**, 5419–5423.

MORRELL, J.I. (1989). Application of *in situ* hybridization with radiactive nucleotide probes to detection of mRNA in the central nervous system. In *Techniques in Immunochemistry*. Academic Press: London, pp. 141–145.

PIEROTTI, M.A., SANTORO, M., JENKINS, R., SOZZI, G., BONGARZONE, I., GRIECO, M., MONZINI, N., MIOZZO, M., HERRMANN, M.A., FUSCO, HAY, I., DELLA PORTA, G. & VECCHIO, G. (1992). Characterization of an inversion on the long arm of chromosome 10 juxtaposing D10S170 and *ret* and creating the oncogenic sequence *ret*/PTC. *Proc. Natl Acad. Sci., USA*, **89**, 1616–1620.

PONT, A. (1979). Secretion and metabolism of calcitonin in man. In De Groot (ed.) *Endocrinology*, Vol. 2. Grune & Stratton: New York, pp. 641–646.

SANTORO, M., ROSATI, R., GRIECO, M., BERLINGIERI, M.T., LUCA-COLUCCI D'AMATO, G., DE FRANCISCIS, V. & FUSCO, A. (1990). The *ret* proto-oncogene is consistently expressed in human pheochromocytomas and thyroid medullary carcinomas. *Oncogene*, **5**, 1595–1598.

SANTORO, M., CARLOMAGNO, F., HAY, I., HERMANN, M., GRIECO, M., MELILLO, R., PIEROTTI, M.A., BONGARZONE, I., DELLA PORTA, G., BERGER, N., PEIX, J.L., PAULIN, C., FABIEN, N., VECCHIO, G., JENKINS, R. & FUSCO, A. (1992). *Ret* oncogene activation in human thyroid neoplasms is restricted to the papillary cancer subtype. *J. Clin. Invest.*, **89**, 1517–1522.

SZENTIRMAY, Z., ISHIZAKA, Y., OHGAKI, M., TAHIRA, T., NAGAO, M. & ESUMI, H. (1990). Demonstration by *in situ* hybridization of *ret* proto-oncogene mRNA in developing placenta during mid term gestation. *Oncogene*, **5**, 701–705.

TAKAHASHI, M., BUMA, Y., IWAMOTO, T., INAGUMA, Y.,. IKEDA, H. & HIAI, H. (1988). Cloning and expression of the *ret* proto-oncogene encoding a tyrosine kinase with two potential transmembrane domains. *Oncogene*, **3**, 571–578.

WILLIAMS, E.D. (1980). Recent results in cancer research. In Duncan, W. (ed.) *Thyroid Cancer*. Springer-Verlag: Berlin, pp. 47–55.